# Urban land use impact on soil heavy metal levels in Lafayette, Louisiana (USA)

**DOI:** 10.1371/journal.pone.0344559

**Published:** 2026-03-18

**Authors:** Holly L. Heafner, Anna A. Paltseva

**Affiliations:** 1 Delta Urban Soils Laboratory, School of Geosciences, University of Louisiana at Lafayette, Lafayette, Louisiana, United States of America; 2 Departments of Agronomy and Horticulture and Landscape Architecture, College of Agriculture, Purdue University, West Lafayette, Indiana, United States of America; Nepal Agricultural Research Council, NEPAL

## Abstract

Urban soils can concentrate metal(loid)s from legacy and ongoing urban activities, yet mid-sized U.S. cities remain under characterized. We quantified As, Cr, Cu, Mn, Ni, Pb, and Zn in 1,290 composite topsoils (0–2.5 cm) across five land uses in Lafayette, Louisiana (USA), and measured pH, Electrical Conductivity (EC), and soil organic matter (SOM). We compared concentrations to local geochemical backgrounds and screening levels and calculated Pollution Index (PI), Pollution Load Index (PLI), Geoaccumulation Index (Igeo), Single Ecological Risk Index (Ei), and Potential Ecological Risk Index (PERI). Metals were highly heterogeneous (e.g., max: Pb = 6,877; Zn = 6,776; Cr = 3,024; Mn = 20,826 mg/kg). Relative to background, exceedance rates were: Zn 99%, Cr 93%, Mn 92%, Pb 83%, Cu 60%, As 54%, Ni 15%. Only 7% of samples exceeded the current USEPA Pb residential soil screening level (200 mg/kg); none exceeded Cu, Ni, Zn, or Mn screening values. Land-use patterns were clear: Industrial > Roads > Residential > Parks/Gardens, with Pb and Zn driving most enrichment. Spearman correlations showed a Pb–Zn–Cu cluster (r up to 0.58) and weak associations between metals and pH, EC, or SOM (|r| ≲ 0.18), indicating limited control of basic soil properties at city scale. PCA and clustering similarly grouped Pb–Zn–Cu, while Ni remained near background. Mean PI values were highest for Pb (6.27) and Zn (6.24); mean PLI = 2.14 (anthropogenic signal), whereas PERI indicated low overall ecological risk (mean = 10.85) with localized hotspots. Results show that Lafayette exhibits a low-to-moderate, legacy-dominated contamination profile, principally Pb and Zn in industrial and roadside settings predominantly in the city center, while parks and gardens are comparatively clean. Findings support targeted risk communication and site-specific management rather than city-wide remediation.

## Introduction

Urban soils play critical roles in maintaining city ecosystem services by supporting vegetation, mitigating flooding and heat island effects, storing carbon, and buffering pollutants [1–4]. Yet these soils are among the most underrecognized components of the urban environment. Heavy metal(loid)s such as cadmium (Cd), chromium (Cr), copper (Cu), mercury (Hg), nickel (Ni), lead (Pb), and zinc (Zn) accumulate in urban soils primarily through fossil fuel emissions, vehicle exhaust, construction materials, and the degradation of legacy contaminants like lead-based paint [5,6]. Once deposited, these metals persist for decades, posing chronic risks to human and ecosystem health.

Beyond atmospheric deposition, additional inputs arise from fertilizers, pesticides, and soil amendments commonly used in agricultural and landscaping practices, which often contain As, Cu, Mn, Ni, and Zn, further contributing to soil contamination [7]. Geogenic processes such as parent material weathering, also contribute metals like As, Cr, and Ni. While these represent natural background levels, anthropogenic activities substantially elevate metal(loid) concentrations in urban settings [5,8].

Industrial operations, vehicular traffic, and mechanical wear release Cu, Ni, Pb, and Zn to the atmosphere, which subsequently deposit into surface soils [2,5,6]. Elevated levels of Cr, Cu, Ni, and Pb are also common near rail infrastructure due to emissions and debris from train operations [5]. In older neighborhoods, legacy Pb contamination remains widespread despite the ban on lead-based paints in 1978, with hotspots strongly linked to property age and historic urban development [9].

Louisiana’s urban soils reflect a distinctive mix of industrial emissions, legacy housing materials, and hydrologic disturbance. Along the Mississippi River Industrial Corridor (“Cancer Alley”), the 135-km stretch between Baton Rouge and New Orleans, dense petrochemical development has generated long-term airborne deposition of metals and other pollutants, producing one of the highest cumulative environmental risk indices in the United States [10]. A statewide assessment by Twumasi et al. (2020) further showed that more than 600 abandoned pits and multiple Superfund sites are concentrated in southern Louisiana, where historical industrial and transportation corridors have contributed to soil contamination dominated by Pb, As, Cd, Hg, and Zn [11]. In New Orleans, multi-decadal monitoring shows that declines in topsoil Pb parallel decreases in children’s blood-lead levels, confirming the soil–health link [12]. Post-Katrina sediment and soil studies also identified As, Pb, and Zn enrichment from flood-redistributed materials [13]. In Baton Rouge, pXRF surveys of schoolyards revealed locally elevated Pb, indicating residual contamination in public play areas [14]. In Lake Charles, Hurricane Laura (2020) triggered industrial accidents and major chemical releases, sharply increasing concerns about acute soil and air pollution exposures in historically marginalized communities already burdened by environmental risk [15]. By contrast, Lafayette, a rapidly growing inland city has not yet been systematically assessed for urban soil metal(loid) contamination. Its mixed residential, commercial, and transportation land uses, combined with moderate traffic density and limited industrial inputs, provide an opportunity to characterize contamination levels and spatial patterns in a mid-sized, non-industrial Louisiana city. Such data can serve as a screening-level evaluation to compare with better-studied coastal urban centers and to inform local risk communication and land-use decisions.

We hypothesized that [1] heavy metal(loid) concentrations vary significantly among land uses, with higher levels in industrial, roadside, and older residential areas; [2] basic soil health properties (pH, SOM, EC) are correlated with metal concentrations; and [3] spatial patterns of contamination reflect Lafayette’s historical development, with potential hotspots posing exposure risks to children. Thus, to test these hypotheses, we assessed heavy metal(loid) concentrations across multiple urban land uses in Lafayette, Louisiana. The objectives of this study were to:

1. Quantify the concentrations and spatial distribution of heavy metal(loid)s (As, Cr, Cu, Mn, Ni, Pb, Zn) across residential, recreational, and industrial sites in Lafayette;
2. Compare these values with national, state soil screening and background geochemical levels to evaluate contamination severity; and
3. Calculate ecological and health risk indices to determine potential exposure risks for residents.

By providing this first soil heavy metal(loid)s dataset for Lafayette, LA, this work contributes to a broader understanding of urban soil contamination dynamics in smaller, less-industrialized cities, supporting informed public-health and urban-planning decisions across Louisiana.

## Methods

### Study area

**Fig 1:**
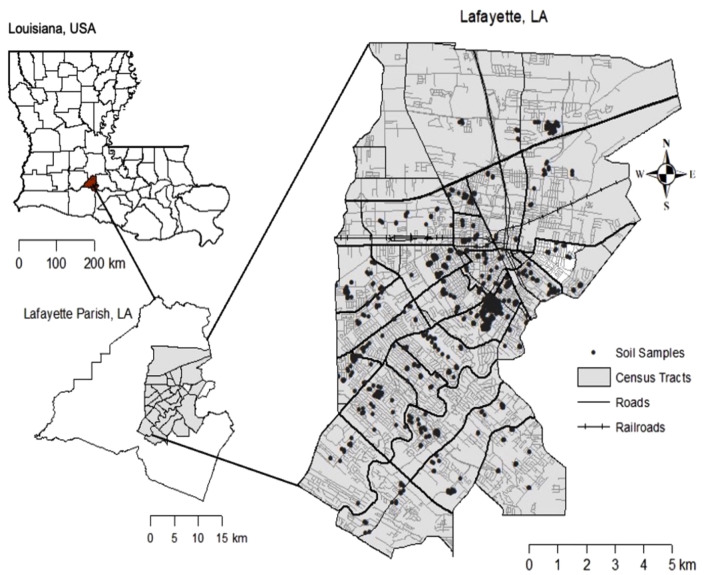
Map of the study area showing 1,290 composite topsoil samples within 24 census tracts of Lafayette, LA.Republished from Heafner and Paltseva (2024) [19] under a CC BY license, with permission from Springer, original copyright 2024.

Lafayette, Louisiana (Fig 1), lies in the south-central region of the state, approximately 50 km north of the Gulf Coast. The city covers an area of 144 km^2^ within Lafayette Parish, which spans more than 700 km^2^ [16]. The region’s soils primarily derive from Pleistocene-aged sandstones, which serve as the predominant parent material. Alfisols are the prevailing soil order, typically featuring a dense claypan horizon that influences drainage and root penetration. Much of the landscape also contains alluvial sediments overlain by younger loess deposits [17]. According to the USDA Web Soil Survey, Lafayette’s soil textures range from heavy clays to loams and fertile silt loams, reflecting the area’s varied topography of flat plains, gently rolling uplands, and low-lying deltaic zones prone to flooding [18].

Lafayette Parish was officially established in 1823, following the settlement of the Acadians around 1770 [20]. Since the late 19th century, Lafayette has experienced considerable population growth, becoming the fourth largest city in Louisiana, with a current population of approximately 120,000 residents [21]. The initial inhabitants, the Attakapas, primarily engaged in livestock grazing, fishing, and hunting, rather than agriculture [22]. In the early 18th century, European settlers mainly participated in fur trading and trapping. However, by the late 1800s, with the extension of the railroad and the influx of European farmers, large-scale agriculture, particularly the cultivation of sugarcane, rice, and cotton, began to play a crucial role in shaping the local economy [17]. During this period, nearly half of Lafayette’s population comprised enslaved African Americans, who were primarily employed on plantations. Post-Civil War, agricultural practices continued to exploit both freed African Americans and small White farmers through sharecropping and tenant farming systems [23]. While agriculture remains a significant aspect of Lafayette’s economy, the discovery of extensive petroleum reserves in the early 1900s shifted the economic focus toward the oil and gas industry, which has since become the predominant economic driver [20].

### Soil sampling and community engagement

From 2021 to 2023, a total of 1,290 composite topsoil samples were systematically collected to represent the 24 core census tracts within Lafayette, LA. Composite topsoil samples (0–2.5 cm) were collected, representing the primary zone of contact where children are most likely to interact with bare soil surfaces [24]. Heavy metal(loid)s tend to concentrate in this uppermost layer, existing in various forms such as dissolved ions, precipitates, organically bound complexes, or exchangeable ions adsorbed onto mineral surfaces [25,26]. For the general population, ingestion of contaminated soil particles is a key exposure route; however, for children, frequent hand-to-mouth activity makes ingestion the dominant pathway for Pb and other toxic metals [27,28]. Accordingly, this study prioritized sampling locations with elevated potential for child exposure, including residential lawns, building perimeters, and public play areas. This study did not involve human participants or the collection of identifiable personal data. Soil samples were collected from public spaces or with permission from landowners. Therefore, institutional ethics approval was not required.

For each site, metadata, including GPS coordinates, a site photograph, and descriptive information, were recorded via smartphone. Site descriptions specified whether samples were obtained from (a) public areas, (b) residential zones, (c) busy roadways, (d) current or former industrial sites, (e) building driplines or foundations, (f) street sides, (g) open greenspaces such as parks or vacant lots, (h) unmanaged garden soils, (i) compost-amended or otherwise managed soils, (j) raised garden beds, (k) children’s play areas, (l) near painted structures, or (m) locations with visible paint debris [29].

In each census tract, 15 composite soil samples were collected, with each composite consisting of at least five thoroughly mixed subsamples. In residential areas, samples were collected within 1 meter of house foundations (n = 225), street sides (n = 225), and from open spaces such as lawns or greenspaces (n = 313) [30,31]. Additional samples were taken from home gardens (n = 62), community gardens (n = 44), public areas including busy roadways (n = 35), public parks (n = 288), and industrial or commercial zones (n = 43). Note, due to limited sample material, only 981 samples were available for pH and EC analysis.

Sampling locations were chosen based on property access and size. Larger areas, like vacant lots or fields in public parks, employed a grid sampling method to account for soil heterogeneity. Due to the high variability in heavy metal distribution across urban soils, influenced by land use history and soil properties, sample numbers were adjusted by area, typically with one composite sample per 100 m² [3,4,32]. With authorization from the Lafayette Consolidated Government Development and Planning, soil samples were collected from public locations, including city-managed parks. Additional permission was obtained from Moncus Park for sampling within its grounds. However, uniform sampling throughout the city was constrained by limited access to private properties and industrial sites, as well as a shortage of available researchers. As a result, sampling at private residences depended largely on the willingness of residents to participate.

To overcome these challenges, a comprehensive community outreach campaign was launched, modeled after the VegeSafe program in Australia [33]. This campaign utilized local media, social media, and direct outreach at neighborhood meetings and through volunteer organizations. Clear sampling instructions were distributed, and collection stations were established at community events. This strategy successfully engaged over 100 residents, who contributed soil samples, thereby enhancing the representation of private properties in the study. For more detailed descriptions of the methods and engagement strategies used, see Heafner & Paltseva, 2024.

### Soil properties and elemental analyses

Laboratory analyses of the soil samples were conducted at the Delta Urban Soils Laboratory, University of Louisiana at Lafayette. Soil organic matter (SOM) content, expressed as a percentage of the soil sample’s dry weight, was determined using the loss on ignition (LOI) method. Samples were first oven-dried at 105 °C to constant weight, then combusted at 550 °C for 20 min to remove organic matter after grinding with a mortar and pestle and sieving to <2 mm particle size with a sieve #10. Soil pH was determined using a 1:1 soil-to-deionized (DI) water slurry and measured with a Hanna Instruments HI5522 pH meter equipped with a conic probe. Electrical conductivity (EC) was measured using a YSI 3100 conductivity meter with a 1:2 soil-to-DI water ratio. Quality control was ensured using standard reference materials (SRMs), including buffer solutions of pH 4.01, 7.01, and 10.01, and a 1413 μS cm ⁻ ¹ conductivity standard. Both probes were calibrated with DI water before each analytical session to ensure accuracy and consistency.

Elemental analysis of heavy metal(loid)s such as As, Cd, Cr, Cu, Hg, Mn, Ni, Pb, and Zn was performed utilizing a handheld XRF analyzer in a benchtop stand (Thermo Niton XL3t 955 Ultra). First, samples were dried in the oven at 105°C for at least 24 hours, followed by grinding and sieving to <2 mm particle size [34]. The soil samples then were placed into low-density polyethylene bags in which they were analyzed by the XRF for 90 seconds each. Standard reference materials (SRM 2710a, SRM 2711a, SRM 2704, and SRM 2709a) were used with each batch of samples, and the experimental values were within 100 ± 10% of the certified values, indicating good analytical precision and acceptable accuracy for environmental soil matrices. Previous studies have demonstrated comparable accuracy of pXRF measurements for soil heavy metals in heterogeneous urban matrices, supporting its reliability for rapid screening and spatial assessments [35–37]. The detection limit of the instrument in SRM matrix is 7 mg/kg for As, 12 mg/kg for Cd, 30 mg/kg for Cr, 15 mg/kg for Cu, 9 mg/kg for Hg, 65 mg/kg for Mn, 30 mg/kg for Ni, 8 mg/kg for Pb, and 12 mg/kg for Zn, according to the manufacturer [38]. Measurements resulting in a reading of less than the limit of detection (LOD) were substituted with the corresponding limit for statistical purposes.

### Environmental risk indices

Indices of soil heavy metal contamination can be useful in determining and predicting the magnitude and severity of anthropogenic pollution [1,39,40]. To assess contamination levels in Lafayette’s urban soils, the (PI), pollution load index (PLI), geoaccumulation index (Igeo), single ecological risk index (Ei), and potential ecological risk index (PERI) were calculated.

The PI is the ratio of a heavy metal’s measured concentration (Ci) to the corresponding metal’s geochemical background concentration (Bi). Studies also term this ratio contamination factor or enrichment factor, each determining the anthropogenic influence on soil pollution in comparison with the natural background levels, or pre-industrial levels [41,42]. The following intervals define the results of the PI calculation: PI < 1 – low contamination, 1 ≤ PI < 3 – moderate contamination, 3 ≤ PI < 6 – considerable contamination, and PI ≥ 6 – very high contamination from anthropogenic sources.


PIi=Ci/Bi

The PLI incorporates contamination caused by multiple different heavy metals present, giving an overall estimation of soil pollution severity. This index is calculated using the geometric mean of PI values for the number of metals (n) [43,44]. PLI values >1 indicate anthropogenic pollution, while PLI values near 1 indicate metal levels congruent with the natural background.


PLI= (PI1 x PI2 x...PIn)1/n

The geoacummulation index (Igeo) compares the measured concentration of a metal with its background concentration, but also accounts for possible variations in the parent material of a soil with a factor of 1.5 [45]. The Igeo was defined by six intervals: Igeo≤0 – unpolluted, 0 < Igeo≤1 – unpolluted to moderately polluted, 1 < Igeo≤2 – moderately polluted, 2 < Igeo≤3 – moderately to strongly polluted, 3 < Igeo≤4 – strongly polluted, 4 < Igeo≤5 – strongly to extremely polluted and Igeo>5 – extremely polluted [45].


Igeo=log2[Ci/(1.5Bi)]

The ecological risk index (Ei) and the potential ecological risk index (PERI) were developed by Hakanson (1980) and incorporate the toxicity of heavy metals exposure into the risk assessment indices by way of toxic response factors for each metal (Ti) [41]. The Ti for each metal evaluated in the present study are as follows: As=10, Cd = 30, Cr = 2, Cu = 5, Mn = 1, Ni = 5, Pb = 5, and Zn = 1. The resulting Ei is defined as: Ei < 40 – low risk, 40 ≤ Ei < 80 – moderate risk, 80 ≤ Ei < 160 – considerable risk, 160 ≤ Ei < 320 – high risk, and Ei ≥ 320 – very high risk.


$${\text{E}}_{\text{i}}\;={\text{T}}_{\text{i}}\text{ x }{\text{PI}}_{\text{i}}$$

Utilizing the results of Ei calculations for each metal, PERI estimates the overall risk to the environment by the level of heavy metal pollution present. This index is the sum of Ei for each heavy metal, defined by intervals: PERI<150 – low risk, 150 ≤ PERI<300 – moderate risk, 300 ≤ PERI<600 – considerable risk, and PERI>600 – very high risk.


PERI = ∑i=Ei

### Statistical and geospatial analysis

Descriptive statistics, such as the range, central tendency, and variability measures, were calculated for each heavy metal(loid) such as As, Cr, Cu, Mn, Ni, Pb, and Zn. Due to minimal detections above the LOD for Cd and Hg using a portable X-ray Fluorescence (XRF) analyzer, these metals were excluded from further statistical analysis. Only 1% (n = 16) of samples exceeded the LOD of 12 mg/kg for Cd, and no samples exceeded the LOD of 9 mg/kg for Hg. Relationships between heavy metal(loid) concentrations and land use categories were assessed using Spearman’s rank correlation coefficients, with a significance level of p < 0.05. In RStudio 2023.12.1 using R 4.2.3 we also performed a factor analysis on the dataset to identify underlying patterns in metal concentrations across different land uses, retaining two factors based on eigenvalue criteria and interpretability, and applied a varimax rotation to maximize the clarity of factor loadings [46,47]. The hierarchical clustering method was used for creating the dendrogram. And Principal Component Analysis (PCA) circle plot helped visualize the underlying relationships between the variables, giving insights into soil quality and potential contamination patterns.

The spatial distribution of soil heavy metal concentrations in Lafayette was mapped using ESRI ArcMap 10.8.2. Central coordinates for each composite soil sample were recorded. The kriging method, a spatial analysis tool, was applied to interpolate metal concentrations and identify patterns and hotspots [4,48,49].

To evaluate the severity of soil pollution in Lafayette, measured soil heavy metal concentrations were compared with national soil screening levels given by the USEPA and Louisiana Department of Environmental Quality (LDEQ) for residential soils [50,51]. The geochemical background level of soil metal(loid)s in the Lafayette region were also used for comparison. Background samples were collected by the Louisiana USDA National Resource Conservation Service (USDA NRCS) team at a depth of 0–10 cm with an auger and analyzed with the portable XRF analyzer at the Delta Urban Soils Laboratory at the University of Louisiana at Lafayette. Reference values for soil heavy metal(loid)s utilized in the present study are listed in S1 Table.

## Results

### Soil pH, EC, and organic matter content

In this study, we analyzed the basic soil health properties to understand soil characteristics and variability across different locations in Lafayette, LA (Table 1). For 981 samples, the soil pH ranged from 4.4 to 8.8, with a mean of 6.5. For 1,114 samples measured for SOM via loss on ignition, the percentage of SOM ranged from 2% − 45%, with a mean of 10%. Soils (n = 981) tested for EC resulted in a range of 0.07–19.60 µS/cm, classifying Lafayette’s urban soils as non-saline. Overall, the soil properties of Lafayette’s urban areas indicate a predominance of slightly acidic to neutral soils with a medium range of soil organic matter.

**Table 1:** Soil health parameters in public and residential areas in Lafayette, LA.

Land Use	pH	Salts, µS/cm	OM, %
**Busy roads** (n = 35)			
Mean	7.01	307	9.2
Min	5.97	79	3.9
Max	7.89	1392	17.5
**Industrial** (n = 43)			
Mean	6.99	207	8
Min	5.82	52	4.1
Max	7.66	329	11.7
**Parks** (n = 288)			
Mean	6.52	382	9.7
Min	4.45	42	1.7
Max	8.83	1899	31.5
**Residential** (n = 763)			
Mean	6.44	291	10
Min	4.40	7	1.9
Max	8.5	1960	45
**Gardens** (n = 106)			
Mean	6.41	278	11.4
Min	5.32	43.5	2.7
Max	7.39	1219	44.4

### Soil heavy metal(loid)s

#### Concentrations and distribution.

The summary statistics for soil heavy metal(loid)s of 1,290 topsoil samples from urban soils of Lafayette, LA, indicate significant variability in metal concentrations across different sampling locations (Table 2). The concentrations of heavy metal(loid)s in Lafayette’s urban soils showed wide variation across the sampling sites (Table 2). Overall, Pb and Zn displayed the largest ranges and highest variability, with extreme values far exceeding the medians, indicating localized enrichment hotspots. Other metal(loid)s also exhibited moderate to high dispersion, while Ni was comparatively uniform, suggesting a mainly geogenic origin. All elements showed positive skewness, confirming right-tailed distributions dominated by a few highly contaminated sites.

**Table 2:** Summary statistics of urban soil heavy metal(loid)s’ concentrations (mg/kg) in Lafayette, LA (N = 1,290).

Element	Min	Q25	Median	Q75	Max	Mean	GM	SD	CV (%)	Skewness
**As**	<7	7	8	11	263	11	9	10	95	14
**Cr**	<30	56	65	72	3,024	70	63	111	157	22
**Cu**	<15	17	24	33	838	33	26	42	128	11
**Mn**	<65	404	533	683	20,826	574	517	605	105	29
**Ni**	<30	30	30	30	87	32	–	6	18	4
**Pb**	<8	17	27	61	6,877	88	35	330	373	13
**Zn**	<12	75	107	194	6,776	237	134	488	206	7

Note:

GM: geometric mean.

Cd: 1% (N=16) samples range from <12-20 mg/kg.

Hg: All samples below XRF limit of detection.

Table 3 shows the mean and range of heavy metal(loid) concentrations, and the number of samples exceeding geochemical background concentrations and screening levels. The median and geometric mean values for all heavy metal(loid)s, except Ni, exceeded the naturally occurring, geochemical background levels. Only 15% of samples (n = 195) exceeded the background concentration for Ni, while 54% (n = 695), 93% (n = 1,200), 60% (n = 776), 92% (n = 1,191), 83% (n = 1,078), and 99% (n = 1,277) exceeded the background for As, Cr, Cu, Mn, Pb, and Zn, respectively (Table 2).

**Table 3:** Percentage of urban soil samples (n = 1,290) in Lafayette, LA exceeding USEPA [50], LDEQ soil screening levels [51], and local geochemical background levels for heavy metal(loid)s (mg/kg).

Element	GM	Range	USEPA (% above)	LDEQ (% above)	Bkgrd (% above)
**As**	11	<7-263	0.68 (100)	12 (20)	7 (54)
**Cr**	70	<30−3,024	0.3 (100)	23 (100)	41 (93)
**Cu**	33	<15-838	3,100 (0)	310 (0)	20 (60)
**Mn**	574	<65−20,826	1,800 (0)	–	284 (92)
**Ni**	32	<30-87	820 (0)	160 (0)	35 (15)
**Pb**	88	<8−6,877	200 (7)	400 (3)	14 (83)
**Zn**	237	<12−6,776	23,000 (0)	2,300 (0)	37 (99)

Note:

GM: geometric mean.

USEPA: Soil screening level (2024).

LDEQ: Soil screening level (2003).

Bkgrd: Lafayette geochemical background (samples collected by USDA NRCS, measured at the ULL Delta Urban Soils Lab).

In regard to the soil screening levels determined by the USEPA [50], all samples exceeded the limit for As and Cr, while no samples exceeded the limit for Cu, Mn, Ni, and Zn (Table 3). All of the soil samples exceeded the 23 mg/kg LDEQ [51] screening level for soil Cr. For As, 20% of samples (n = 262) exceed the LDEQ screening level of 12 mg/kg. No samples exceed the 2,300 mg/kg limit for Zn, nor the 160 mg/kg limit for Ni, and less than 1% of samples (n = 3) exceed the 310 mg/kg limit for Cu as set by the LDEQ [51]. According to the USEPA [50] screening level for soil Pb, which was reduced to 200 mg/kg from 400 mg/kg in January 2024, only 7% (n = 89) exceed (Table 2). The LDEQ [51] still uses 400 mg/kg as the soil screening level for soil Pb, of which 3% (n = 40) samples exceed (Table 2).

**Fig 2:**
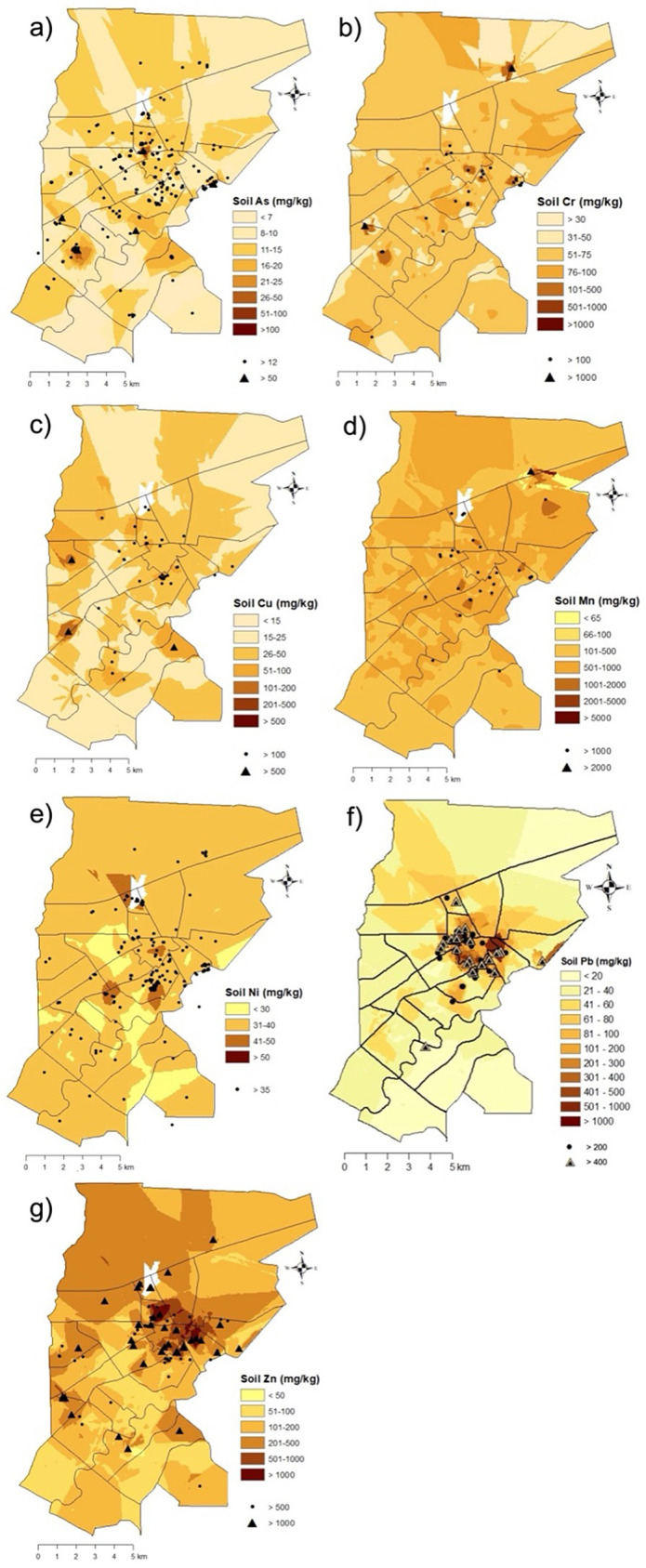
Spatial interpolation maps for urban soil heavy metal(loid)s a) arsenic (As), b) chromium (Cr), c) copper (Cu), d) manganese (Mn), e) nickel (Ni), f) lead (Pb), g) zinc (Zn) in Lafayette, LA (n = 1,290).

Interpolation maps of heavy metal(loid) concentrations are shown in Fig 2, with darker shades indicating higher concentrations. Both metals Pb and Zn (Fig 2f, g) show the highest concentrations clustered in the urban core and around known industrial areas, reflecting strong anthropogenic influence, proximity to traffic corridors, and legacy industrial emissions. Hotspots for Cr, Cu, and Mn (Fig 2b, c, d) coincide with Lafayette’s central and northeastern commercial/industrial districts, as identified in city zoning and development maps, indicating localized influence from historical and ongoing industrial activities. Arsenic and Ni (Fig 2a, e) exhibit lower, more diffuse concentrations, with only a few small hotspots, suggesting a stronger influence of natural geologic background or more limited point-source contamination. For all elements, highest concentrations generally occur in the central and northeastern parts of urban Lafayette, corresponding with historic industrial and high-traffic districts. Peripheral and suburban areas consistently show lower metal concentrations for all species, indicating reduced exposure to anthropogenic sources. Overall, these spatial trends confirm that Lafayette’s soil heavy metal contamination is most significant in historic urban-industrial zones, with Pb and Zn showing the most pronounced urban hotspots—patterns that align with known sources such as legacy leaded gasoline, older infrastructure, and industrial emissions.

#### Urban land uses.

**Fig 3:**
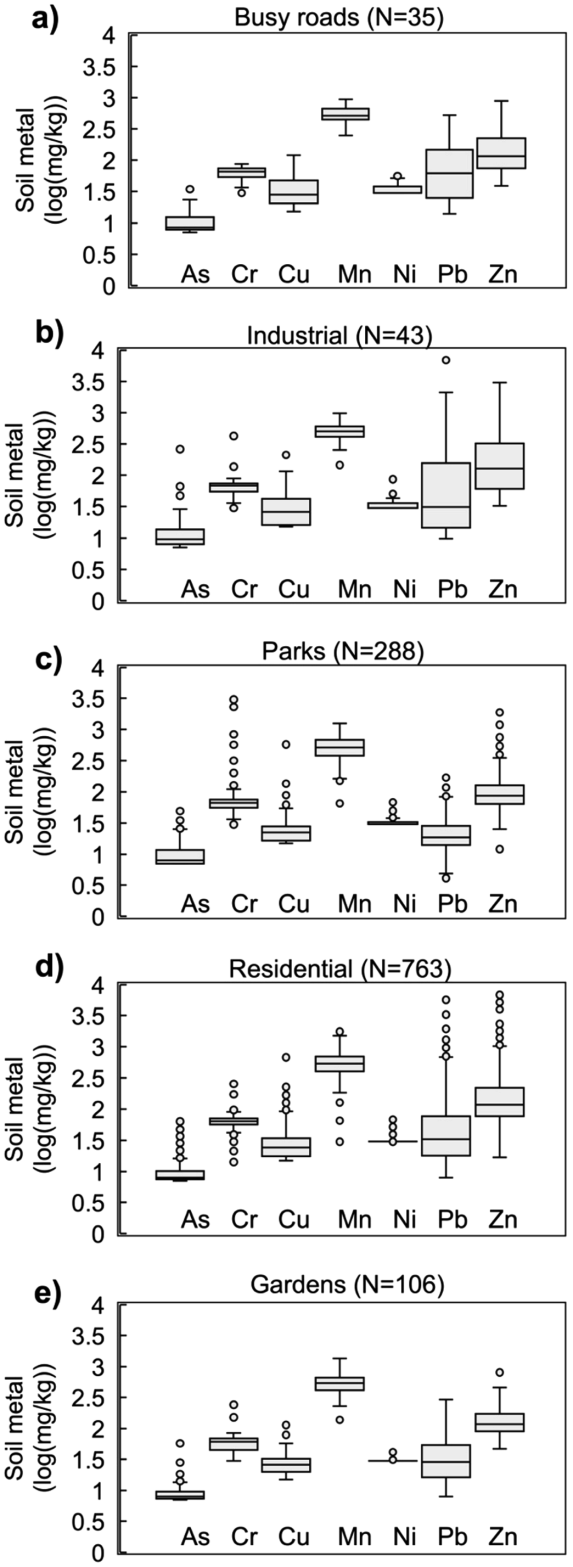
Heavy metal(loid)s for different land uses in Lafayette, LA.

Heavy metal(loid) concentrations differed markedly among urban land uses (Fig 3, S2 Table). Industrial areas contained the highest overall metal loads, particularly for Pb and Zn, and also exhibited the widest variability, consistent with legacy emissions and mixed industrial activities. Roadside soils showed elevated Pb and Zn levels above background values, reflecting contributions from vehicle traffic and road dust. Residential soils displayed moderate contamination, with high variability in Pb and Zn suggesting localized enrichment near older housing and painted structures. Parks and green spaces generally had lower metal concentrations but greater variability in Cr and Mn in parks. Garden soils exhibited the lowest mean concentrations for most elements and comparatively low variance, indicating limited anthropogenic inputs and possible benefits from organic management. Across all land uses, Ni concentrations remained near geogenic background levels. These patterns collectively underscore the strong influence of urban activity type and land management history on spatial metal distribution in Lafayette.

#### Multivariate analyses of metal concentrations.

The Spearman correlation coefficients between heavy metal(loids) concentrations and soil characteristics (n = 948) identified several significant relationships (Table 4). The most significant correlations indicate that Pb, Zn, and Cu are closely linked, likely due to common urban sources such as traffic and industrial activities p < 0.05. The moderate correlations of Cr with As and Pb suggest additional contamination from industrial sources p < 0.05. pH, EC, and OM exhibited only minor influence on metal concentrations—OM was weakly negatively correlated with As, Cr, and Ni, while EC was inversely related to pH.

**Table 4:** Spearman correlation coefficients between heavy metal(loid) concentrations and soil characteristics (n = 948). Asterisks (*) denote statistically significant correlations at p < 0.05.

	As	Cr	Cu	Mn	Ni	Pb	Zn	pH	OM	EC
**As**	1									
**Cr**	0.38*	1								
**Cu**	0.29*	0.32*	1							
**Mn**	0.11*	0.2*	0.29*	1						
**Ni**	0.17*	0.21*	0.2*	0.05	1					
**Pb**	0.21*	0.32*	0.36*	0.37*	0.09*	1				
**Zn**	0.21*	0.19*	0.49*	0.34*	0.04	0.58*	1			
**pH**	0.05	0.01	0.1*	0.02	0.08*	0.01	0.03	1		
**OM**	−0.11*	−0.18*	−0.06	0.07*	−0.14*	−0.03	0.02	0	1	
**EC**	−0.07*	−0.06*	0.01	0.09*	0.02	−0.04	0.03	−0.31*	−0.01	1

The factorial analysis (Table 5) showed that industrial areas have the most substantial overall impact on metal concentrations across both factors, indicating significant metal presence, likely due to various industrial processes and waste. Foundations of houses are strongly associated with Factor 1, suggesting specific metals prevalent in areas with construction materials or activities. Busy roads show a moderate association with Factor 2, likely due to vehicular emissions and road materials. Gardens, playgrounds, residential samples (open spaces and street sides) generally show negative associations with both factors, indicating lower metal concentrations or different metal profiles compared to industrial or foundation areas.

**Table 5:** Factor loadings of metal concentrations across different land uses.

Land Use	Factor 1	Factor 2
**Busy roads**	− 0.084	0.097
**Industrial**	0.029	0.913
**Foundation**	0.376	0.044
**Open space**	− 0.089	0.011
**Street side**	− 0.065	− 0.065
**Gardens**	− 0.185	− 0.095
**Playgrounds**	− 0.238	− 0.054

**Fig 4:**
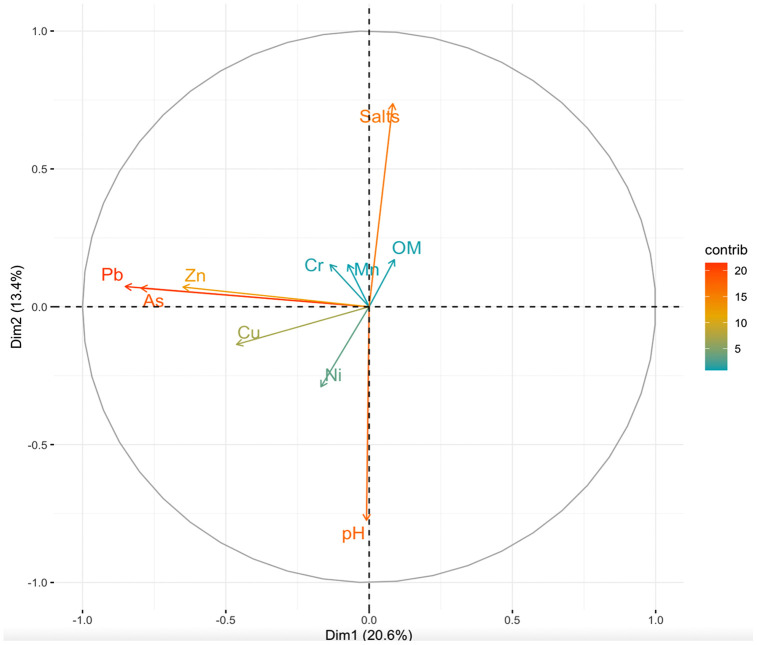
Principal Component Analysis (PCA) biplot of heavy metal concentrations and soil properties in urban soils of Lafayette, LA (n = 1,290).

To explore relationships among metals and soil properties, a PCA was performed on standardized variables (Fig. 4). The first two components explained 34% of the total variance (Dim1 = 20.6%, Dim2 = 13.4%). Dim1 primarily represented a gradient of anthropogenic influence, with high loadings for Pb, Zn, and As, which were closely aligned and contributed most strongly to this axis. These elements are commonly associated with urban traffic, industrial emissions, and legacy paint, consistent with their strong intercorrelations in Table 4. Dim2 captured variability related to soil health conditions, particularly pH and EC. The proximity of Pb, Zn, and As vectors in the biplot indicates a shared contamination source, whereas Ni and Cr were positioned separately, reflecting mixed or partially geogenic origins. Overall, the PCA confirms that urban-derived metals (Pb, As, Zn) dominate the variability in Lafayette’s soils, while intrinsic soil factors contribute less strongly to spatial differentiation.

**Fig 5:**
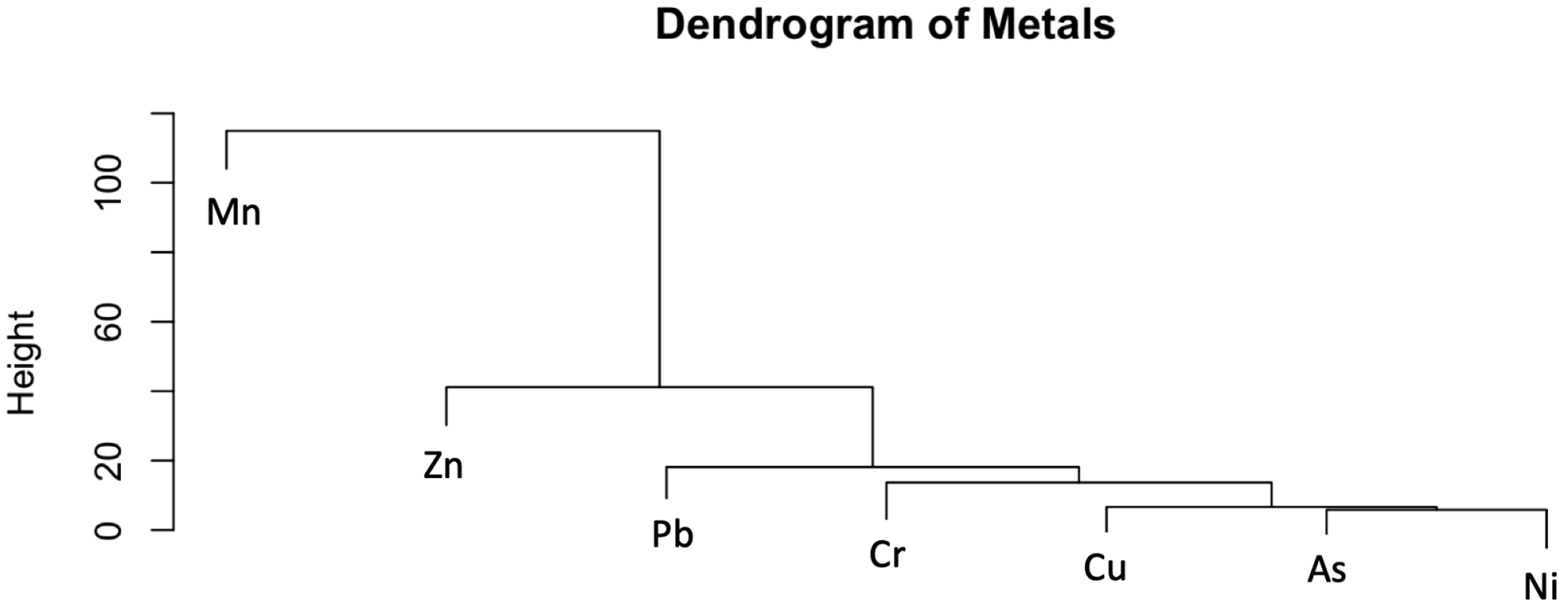
Dendrogram showing the hierarchical clustering of heavy metal(loid)s in urban soils of Lafayette, LA (n = 1,290).

The dendrogram (Fig 5) illustrates the hierarchical clustering of heavy metals based on their similarities in urban soils. It shows that Mn is the most distinct metal, showing the highest separation from the others, suggesting it behaves differently. Zinc and Pb are more closely related and clustered together, indicating similar sources such as historic use of leaded gasoline and vehicles. Chromium, Cu, As, and Ni form another group with even closer associations, suggesting shared origins from industrial emissions, metal processing, or historical use in manufacturing and construction. Both methods indicate that Mn, Zn, and Pb are distinct in their contribution to environmental variation. Fig 5 dendrogram also places Cr and Ni closer to the geogenic side of the clustering, confirming their soil origin rather than anthropogenic enrichment.

#### Environmental risk assessment.

**Fig 6:**
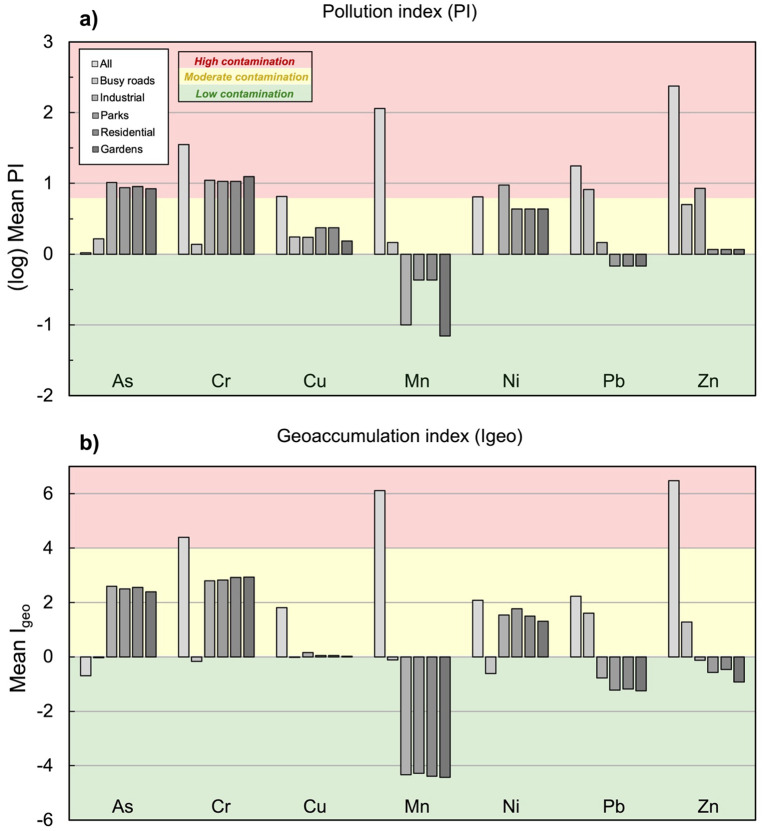
Heavy metal(loid) indices of a) soil pollution (PI) and b) geoacummulation (Igeo) with ranges of uncontaminated (green), moderately contaminated (yellow), and heavily contaminated soil (red) for land uses.

Calculations of environmental pollution indices for metal(loid)s As, Cr, Cu, Mn, Ni, Pb, and Zn revealed high variability for urban soil contamination in Lafayette, LA (Table 6, Fig 6).

**Table 6:** Pollution indices for urban soil heavy metal contamination in Lafayette, LA (n = 1,290).

Index	Mean	Median	Min	Max	SD
**Pollution index (PI)**					
As	1.48	1.10	0.81	37.0	1.41
Cr	1.54	1.42	0.65	66.3	2.43
Cu	1.62	1.19	0.72	41.5	2.07
Mn	1.54	1.43	0.17	55.8	1.62
Ni	0.93	0.87	0.85	2.51	0.17
Pb	6.27	1.94	0.55	487.7	23.4
Zn	6.24	2.83	0.32	178.3	12.9
*Pollution load index (PLI)*	*2.14*	*1.44*	*0.52*	*53.4*	*2.49*
**Geoacummulation index (Igeo)**					
As	−0.70	−0.94	−1.38	4.13	0.60
Cr	4.39	4.43	3.31	9.98	0.48
Cu	0.95	1.68	0.95	6.80	0.78
Mn	6.11	6.15	3.12	11.4	0.62
Ni	2.08	2.0	1.98	3.53	0.21
Pb	2.22	1.86	0.04	9.84	1.53
Zn	6.48	6.16	3.0	12.1	1.26
**Single ecological risk index (Ei)**					
As	14.82	11.02	8.10	369.83	14.15
Cr	3.09	2.84	1.3	132.65	4.86
Cu	8.09	5.97	3.59	207.51	10.36
Mn	7.69	7.13	0.87	278.95	8.11
Ni	4.65	4.34	4.26	12.53	0.85
Pb	31.35	9.68	2.73	2,438.62	117.09
Zn	6.24	2.83	0.32	178.3	12.85
*Potential ecological risk index (PERI)*	*10.85*	*6.26*	*3.02*	*516.91*	*24.04*

#### The Pollution Index (PI).

The pollution index (PI), as shown in Fig 6a, for each metal compares measured concentrations to their corresponding background concentrations, demonstrating high pollution (PI ≥ 6) levels for Pb and Zn with means of 6.27 and 6.24, respectively. These two metals also had the highest maximum PI at 487.7 for Pb and 178.3 for Zn (Table 6). According to PI, only Ni demonstrated a low level of pollution (PI < 1), with a mean of 0.93. The lowest maximum PI was also found for Ni at 2.51, indicating levels of Ni pollution ranging from low (PI < 1) to moderate (1 ≤ PI < 3). Mean PI in decreasing order was as follows: Pb (6.27)> Zn (6.24)> Cu (1.62)> Mn (1.54)=Cr (1.54)> As (1.48)> Ni (0.93) (Table 6, Fig 6a). Incorporating the mean PI of each heavy metal(loid), the pollution load index (PLI) is used to differentiate between anthropogenic (PLI > 1) and naturally (PLI ≤ 1) occurring levels of heavy metal(loid)s. For the urban soils of Lafayette, the mean PLI was calculated at 2.14, indicating evidence of anthropogenic pollution. The PLI ranges from 0.52–53.4, demonstrating the highly variable presence of heavy metal(loid)s, particularly Pb and Zn (Table 6).

#### Geoacummulation (Igeo) Index.

The index of geoacummulation (Igeo), shown in Fig 6b, is similar to PI, but accounts for natural variation in urban soils due to parent material or geologic influences. The only metal(loid) classified as unpolluted (Igeo≤0) was As, with a mean of −0.70 (Table 6, Fig 6b). Only Cu was classified as unpolluted to moderately polluted (0 < Igeo≤1), with a mean of 0.95. Concentrations of Ni and Pb showed moderate to strong pollution (2 < Igeo≤3), with means of 2.08 and 2.22, respectively. The highest mean and maximum Igeo was found for Zn at 6.48 and 12.1, respectively, indicating extreme pollution (Igeo>5). After Zn, metals with the highest maximum Igeo were Mn (11.4), Cr (9.98), and Pb (9.84). The following order of decreasing mean Igeo was determined as follows: Zn (6.48)> Mn (6.11)> Cr (4.39)> Pb (2.22)> Ni (2.08)> Cu (0.95)> As (−0.70).

#### Ecological risk (Ei).

Ecological risk was evaluated by calculating 1) the individual risk of each metal(loid) (Ei) and 2) the PERI for all considered metal(loid)s. Soil contamination levels in Lafayette, LA indicated overall low ecological risk (Ei < 40) for all metal(loid)s (Table 6). The highest Ei was determined for Pb, with a mean of 31.35. The highest maximum Ei was also for Pb, at 2,438.62, which indicates very high ecological risk (Ei ≥ 320). Ordered by descending mean enrichment index (Ei), the metal(loid)s are: Pb (31.35)> As (14.82)> Cu (8.09) Mn> (7.69)> Zn (6.24)> Ni (4.65)> Cr (3.09).

#### Potential ecological risk (PERI).

Potential ecological risk was calculated by combining risk levels determined for each individual metal(loid) (Ei). The mean PERI for the urban soils of Lafayette was calculated at 10.85, indicating low potential risk (PERI<150) (Table 6). The range of values for PERI (3.02–516.91) indicate low to considerable (300 ≤ PERI<600) risk that warrants further concern, particularly for Pb which had a maximum Ei of over 2,000. Although localized soil contamination is present, the overall ecological risk for urban soils in Lafayette is generally low.

## Discussion

Urban soil contamination poses a particular threat to public health through ingestion, inhalation, and dermal contact with contaminated soil particles [3,52]. Children are especially vulnerable due to frequent hand-to-mouth activity and higher soil ingestion rates, leading to elevated blood-metal levels and cognitive or developmental effects [5,53]. Identifying contamination hotspots in accessible public spaces such as parks, playgrounds, and residential areas is essential for risk reduction and informed land management [54].

### Influence of soil properties on heavy metal behavior

Correlation analyses between soil pH, EC, OM, and heavy metal(loid) concentrations (Table 4) revealed generally low coefficients (<0.20), indicating that these soil properties exert minimal influence on the spatial distribution of metals at the city scale. Instead, localized anthropogenic inputs, for example, traffic emissions, past industrial activity, or legacy lead paint, likely exert stronger control over the observed distribution patterns. Other studies have also reported a lack of correlation between soil heavy metal(loid) concentrations and physiochemical properties [55–57].

### Soil heavy metal(loid)s

#### Cu, Pb, and Zn.

Lafayette’s downtown area, established in the early 1800s, has accumulated significant quantities of legacy heavy metal(loid)s such as Cu, Pb, and Zn, creating contamination hotspots (Fig 3). Elevated Pb concentrations are linked to Pb-based paint, vehicular emissions, and industrial activity [58–60]. The highest soil Pb concentrations were discovered in a formerly industrial zone (6,877 mg/kg) and near an older home’s foundation (5,659 mg/kg) (Table 3). Extensive research links urban soil Pb contamination to historic Pb-based paint use and leaded gasoline. Lead-based paint was banned in 1978, explaining higher soil Pb near older residential foundations [31,61,62]. Lafayette houses built post-1970 show significantly lower contamination (p < 0.05) near foundations [19]. Census tracts with over 50% of houses built before 1970 report higher Pb levels, with an average of 61 mg/kg compared to an average median of 20 mg/kg in census tracts with fewer pre-1970 homes [19].

In addition to paint deterioration, legacy Pb from leaded gasoline emissions has contributed to contamination near roadways [4,49,60,63]. Despite the 1996 US ban on leaded gasoline in 1996, following the Lead Reduction Act of 1984, Lafayette shows high Pb levels near busy roads in the historic city center (Fig 3, 4f) [63–65]. Traffic remains a key source of heavy metals in urban soils, linked to vehicle part deterioration and exhaust deposition (Ajmone-Marsan and Biasioli, 2010; Mielke et al., 2000). Correlation analyses support this: Pb-Zn (r = 0.58) and Cu-Zn (0.49) are all significantly correlated (p < 0.05) (Table 4), indicating shared sources. Similar correlations and spatial patterns for Cu, Pb, and Zn aligned with anthropogenic sources, elevated levels near city centers, older residential areas, major traffic routes, and industrial sites were found in other studies [49,60,66,67]. Hotspots of Pb and Zn in Lafayette cluster around the city center, older neighborhoods, and former industrial areas (Fig 4f, g).

Only 7% of Lafayette’s samples exceed USEPA’s Pb guideline of 200 mg/kg, with no samples exceeding guidelines for Cu or Zn (Table 2) [50]. However, compared to natural background levels, 83% of samples exceed background Pb (14 mg/kg), 60% exceed Cu (20 mg/kg), and 99% exceed Zn (37 mg/kg), underscoring the importance of using local geochemical backgrounds to assess anthropogenic contamination.

#### As and Mn.

Metals like As and Mn in urban soils originate from both geogenic and anthropogenic sources, depending on geography and human activities [68,69,70]. Industrial emissions and vehicular traffic are common anthropogenic contributors, particularly after the phaseout of leaded gasoline, when methylcyclopentadienyl manganese tricarbonyl (MMT) was introduced as a fuel additive [71,72]. In Lafayette, Mn levels generally follow these trends, though one public park exhibited a pronounced hotspot exceeding 20,000 mg/kg (Table 3). Comparable behavior has been observed in New Orleans, where Mn was abundant in both alluvial and urban soils (max = 705 mg/kg, median 164 in alluvium; max = 1,089 mg/kg, median 138 in urban soils) [72]. Although urban enrichment was limited, spatial patterns revealed elevated Mn concentrations near southwestern industrial zones and minor peaks in the city center. Mn in Louisiana soils largely reflects geogenic inputs from Mississippi River alluvium with moderate anthropogenic influence from MMT-fueled emissions [72].

In southern Louisiana, As contamination stems from several sources, including defunct cattle dipping vats that used As-rich insecticides to protect livestock, arsenical pesticides applied to cotton fields, industrial activities, and historical cemeteries where As-based embalming fluids were used [73]. In Lafayette, while all samples surpass the USEPA’s screening level of 0.68 mg/kg for As, only 54% exceed the local geochemical background (7 mg/kg) (Table 2). A study in Baton Rouge, LA [74] determined urban soil As levels to be largely geogenic, averaging 5 mg/kg, while Lafayette’s mean of 11 mg/kg and max of 263 mg/kg in industrial areas suggests stronger anthropogenic influence.

#### Cr and Ni.

In Lafayette, total soil Cr concentrations exceeded the geochemical background of 41 mg/kg in 93% of samples (n = 1,200). Although XRF analysis cannot distinguish between Cr(III) and Cr(VI), the measured concentrations also surpass the USEPA screening level of 0.3 mg/kg for Cr(VI), suggesting possible anthropogenic enrichment (Table 2). However, the regional soil environment in Lafayette, LA, characterized by neutral to slightly acidic pH and reducing conditions, favors the predominance of trivalent chromium (Cr(III)), which is typically bound to clay minerals and organic matter and derived from natural weathering of Pleistocene sediments [75]. The hexavalent form (Cr(VI)) is generally unstable under such conditions and thus less likely to persist. Elevated Cr concentrations in urban environments are commonly associated with industrial emissions, vehicular activity, and dense population centers [60,76,70].

Both Cr and Ni in Baton Rouge, LA soils were attributed primarily to geogenic origins [74]. Similarly, in Lafayette, only 15% of Ni samples (n = 195) exceeded the geochemical background of 35 mg/kg, with none surpassing the USEPA screening level of 820 mg/kg (Table 2). Comparatively, in New Orleans, LA maximum Ni concentrations reached 1,927 mg/kg (median 9.8 mg/kg) and maximum Cr concentrations reached 205 mg/kg (median 2.1 mg/kg), while in adjacent alluvial soils the maxima were substantially lower, 9.7 mg/kg for Ni and 3.0 mg/kg for Cr, reflecting the strong anthropogenic influence within the city [72] In Mielke et al. (2000a), Cr and Ni were weakly correlated with Pb–Zn and more associated with parent material (alluvial sediments) [72].

### Urban land uses

#### Busy roads.

The concentrations of metal(loid)s in roadside soils from Lafayette, Louisiana, indicate moderate enrichment relative to other urban environments (Table 7). Arsenic (12 mg/kg) and Ni (35 mg/kg) fall within ranges commonly reported for urban background soils, comparable to values in Beijing and Hangzhou, suggesting limited anthropogenic accumulation. Chromium (63 mg/kg) and Cu (35 mg/kg) are similar to concentrations observed in Chinese and European cities, likely reflecting traffic-related sources such as vehicular wear and brake emissions. Manganese (550 mg/kg) appears within the global range (330–650 mg/kg). Lead (116 mg/kg) and Zn (190 mg/kg) are moderately elevated, aligning with concentrations reported for European urban soils but substantially lower than those from highly industrialized sites such as Kavala, Greece [77].

**Table 7:** Urban soil heavy metal(loid)s (mg/kg) in different land use categories of studies worldwide.

City	N	As	Cr	Cu	Mn	Ni	Pb	Zn	References
**BUSY ROADS**									
Lafayette, LA, USA	35	12	63	35	550	35	116	190	*Present study*
64 sites across Europe	–	–	28	48	–	25	106	180	[79]
Kavala, Greece	96	38	240	48	–	77	571	175	[77]
Galway, Ireland*	294	–	–	17	650	22	41	82	[81]
Kielce, Poland	10	1	7	14	330	5	29	87	[82]
Beijing, China	80	8	62	30	–	27	35	92	[80]
Hangzhou, China	45	–	53	39	449	23	70	139	[78]
**INDUSTRIAL**									
Lafayette, LA, USA	43	21	73	37	500	36	326	334	*Present study*
Kavala, Greece	96	23	144	43	–	44	180	115	[77]
London, England (Wolverhampton)	25	–	–	139	–	–	144	368	[83]
Ilawa Glowna, Poland	–	–	208	480	–	–	193	1,438	[84]
Alcala de Henares, Spain	290	7	13	9	159	7	22	28	[85]
Qadissiya, Jordan	31	–	22	3	–	–	55	45	[86]
Ghaziabad, India	15	–	807	295	809	279	225	373	[87]
Hangzhou, China	–	–	–	72	–	–	139	346	[88]
**PARKS**									
Lafayette, LA, USA	288	11	92	26	612	33	24	168	*Present study*
Grand Forks, ND, USA	30	9	24	17	818	24	17	80	[42]
Brno, Czech Republic	61	–	–	17	–	–	27	59	[1]
Galway, Ireland*	200	18	–	88	–	–	328	302	[8]
Palermo, Italy	70	–	34	63	519	–	202	138	[89]
Madrid, Spain	40	7	17	14	249	–	22	50	[90]
Seville, Spain	31	–	39	68	471	22	137	145	[43]
Çanakkale, Turkey	42	–	21	28	475	21	18	58	[91]
Peshawar, Pakistan	85	–	35	20	–	64	22	78	[2]
Beijing, China	–	12	64	35	–	27	36	146	[92]
Hangzhou, China	–	–	–	38	–	–	56	94	[88]
Hong Kong, China	594	–	–	25	–	–	93	168	[93]
Xiamen, China	40	–	14	26	347	8	36	100	[3]
Perth, Australia	35	3	12	82	71	5	253	125	[94]
**RESIDENTIAL**									
Lafayette, LA, USA	763	10	64	34	564	32	105	274	*Present study*
New Orleans, USA*	4,388	–	2	16	134	7	100	146	[30]
Grand Forks, ND, USA	10	9	24	17	784	25	15	82	[42]
London, England (Richmond)	106	–	–	48	–	–	271	179	[83]
London, England (Wolverhampton)	178	–	–	62	–	–	111	240	[83]
Kielce, Poland	10	2	8	8	657	6	34	91	[82]
Moscow, Russia	52	8	98	61	–	27	50	190	[95]
Ghaziabad, India	18	–	24	27	184	92	112	113	[87]
Beijing, China	68	–	60	27	521	60	20	92	[96]
Hangzhou, China	–	–	–	50	–	–	91	211	[88]
Shanghai, China	114	–	91	36	634	36	68	178	[96]
Shenzhen, China	75	–	91	22	256	91	54	81	[96]
**GARDENS**									
Community gardens									
Lafayette, LA, USA	44	8	63	27	614	30	47	175	*Present study*
Connecticut, USA	174	6	15	51	–	12	330	176	[97]
New Orleans, USA*	100	5	31	22	–	15	57	129	[98]
New York City, USA*	564	6	13	35	213	10	102	138	[99]
New York City, USA*	106	8	39	55	–	21	140	169	[100]
Melbourne, Australia	39	8	17	40	201	15	102	218	[101]
Baghdad, Iraq	140	–	4	16	–	31	2	24	[102]
Home gardens									
Lafayette, LA, USA	62	9	56	29	532	31	43	133	*Present study*
Baltimore, USA	50	–	–	78	167	6	1,171	588	[103]
New York City, USA*	197	12	53	89	–	30	632	300	[100]
Melbourne, Australia	395	9	50	49	210	16	204	334	[101]
Ottawa, Canada	50	3	45	13	525	16	65	114	[104]
Toronto, Canada	–	3	34	19	604	–	13	–	[105]
Kielce, Poland	20	3	8	12	616	5	23	107	[82]
*Median									

Overall, Lafayette’s roadside soils exhibit a contamination signature dominated by Pb > Zn > Cu, consistent with legacy vehicle emissions, brake and tire wear, and aged painted infrastructure. The presence of heavy metal(loid)s in roadside soils is particularly pronounced within 5 meters of the road, where contamination levels are significantly higher than in other urban soils [78–80]. Compared globally, Lafayette occupies a moderate contamination tier, higher than northern European sites but significantly lower than heavily trafficked Mediterranean or Asian industrial corridors. Without protective measures like dense vegetation or barriers, atmospheric deposition of heavy metal particles into topsoil occurs unimpeded, exacerbating soil contamination [79]. These findings underscore the need for strategic urban planning and vegetation management to reduce heavy metal exposure in urban environments.

#### Industrial areas.

Industrial soils in Lafayette, LA, exhibit moderate levels of heavy metal(loid)s compared to other urban-industrial environments globally (Table 7). Arsenic (21 mg/kg) and Cr (73 mg/kg) concentrations fall within the lower to mid-range of values reported for European cities such as Kavala, Greece (As = 23 mg/kg; Cr = 144 mg/kg) and Alcala, Spain (As = 7 mg/kg; Cr = 13 mg/kg), and are substantially lower than industrial zones in India, where Cr exceeds 800 mg/kg [87]. Copper (37 mg/kg) and Ni (36 mg/kg) levels are similarly moderate, indicating diffuse urban and natural inputs rather than concentrated metallurgical or electroplating sources. Manganese (500 mg/kg) lies within the typical range of background to slightly enriched values for industrial soils, suggesting limited anthropogenic influence. Lead (326 mg/kg) and Zn (334 mg/kg) are the dominant contaminants, exceeding concentrations typical of residential or park soils but remaining far below levels observed in heavily industrialized sites. Overall, Lafayette’s industrial soils reflect a contamination profile characterized by elevated Pb and Zn linked to legacy urban activities, vehicle emissions, construction materials, and industrial residues, rather than current large-scale industrial discharge. This pattern positions Lafayette within the moderately contaminated category typical of small to mid-sized cities in developed regions, where historical urbanization and transportation remain the primary drivers of soil metal accumulation.

#### Parks.

Urban park soils in Lafayette, LA, show low-to-moderate heavy metal(loid) enrichment compared with other global cities (Table 7). Mean concentrations of As (11 mg/kg), Cr (92 mg/kg), Cu (26 mg/kg), Ni (33 mg/kg), Pb (24 mg/kg), and Zn (168 mg/kg) fall close to or below typical urban park ranges reported in Europe and Asia. For example, As levels are comparable to Grand Forks, USA (9 mg/kg) [42] and Madrid, Spain (7 mg/kg) [90], but well below those in Galway, Ireland (18 mg/kg) [8]. Chromium in Lafayette (92 mg/kg) is slightly higher than in Beijing (64 mg/kg) [106] or Seville (39 mg/kg) [43], yet markedly lower than the 251 mg/kg average observed in Palermo, Italy [89]. Copper and Zn concentrations in Lafayette parks (26 mg/kg and 168 mg/kg) are moderate and align with reported values for Hong Kong (25 mg/kg Cu, 168 mg/kg Zn) [107], indicating diffuse urban deposition rather than strong point sources. Lead levels (24 mg/kg) are among the lowest globally, an order of magnitude lower than Galway (328 mg/kg) or Seville (137 mg/kg), suggesting limited legacy lead contamination in Lafayette’s green spaces. Manganese (612 mg/kg) remains within natural background ranges found in U.S. and Mediterranean park soils, reflecting geogenic rather than anthropogenic control. This reduced level of contamination can be largely attributed to the parks’ origins; many were developed on previously agricultural land rather than industrial sites, which inherently posed a lower risk of contamination from heavy metals [108]. Taken together, these values indicate that Lafayette’s park soils are relatively clean in a global context. These findings also align with regional Louisiana patterns showing reduced metal burdens in managed recreational spaces compared to residential and roadside environments [109].

#### Residential areas.

The concentrations of metal(loid)s in Lafayette’s residential soils are within the range reported for other urban environments but show element-specific variations reflecting both local geology and anthropogenic influence (Table 7). Arsenic (10 mg/kg) is comparable to values in Grand Forks, USA (9 mg/kg) [42] and Moscow, Russia (8 mg/kg) [95], but much lower than those reported for Shanghai and Shenzhen, China (both 91 mg/kg) [96], suggesting limited industrial input. Chromium (64 mg/kg) ranks among the highest observed, exceeded only by Moscow (98 mg/kg) and Shanghai (91 mg/kg), indicating a combination of geogenic and anthropogenic sources (e.g., pesticides, treated wood). Copper (34 mg/kg) aligns with concentrations in Beijing (27 mg/kg) and Shanghai (36 mg/kg), consistent with moderate enrichment from traffic-related emissions. Manganese (564 mg/kg) is within the global background range, similar to Beijing (521 mg/kg) and Shanghai (634 mg/kg), implying lithogenic control. Nickel (32 mg/kg) exceeds values in New Orleans (7 mg/kg) [109]and Kielce, Poland (6 mg/kg) [82], but remains below those in industrial regions such as Ghaziabad, India (92 mg/kg) [87]. Lead (105 mg/kg) and Zn (274 mg/kg) are moderately elevated, comparable to New Orleans (Pb = 100 mg/kg; Zn = 146 mg/kg) but lower than London (Pb = 271 mg kg; Zn = 240 mg/kg) [83], reflecting legacy contamination from traffic and leaded paint deposition. Overall, Lafayette’s metal profile represents moderate enrichment typical of mid-sized cities dominated by diffuse vehicular and atmospheric inputs rather than heavy industrial activity.

#### Gardens.

Urban garden soils in Lafayette exhibited low variance and mean concentrations of As, Cr, Ni, and Zn (Table 3), with no samples exceeded the LDEQ’s Pb screening level (400 mg/kg) and only one sample exceeded the USEPA’s (200 mg/kg) [50,51]. These findings align with studies like [110] in Adelaide, Australia, where contamination remained low, except near industrial zones. Similarly, [97] found that 36% of Connecticut gardens (n = 25) had at least one sample exceeding the Pb guideline, while 20% had one exceeding the As guideline [97]. Although concentrations of Cu and Zn were elevated above natural background levels, they remained below guideline values, and concentrations of Cd, Cr, and Ni were close to background levels.

For 54 community gardens tested in New York City, only 10% of samples exceeded the Pb guideline [99]. In Los Angeles, CA, 64 soil samples from 11 community gardens exceeded the CA residential guideline of 80 mg/kg for Pb [111]. A study of 27 community gardens in New Orleans found at least one sample per garden exceeded the screening levels for Cd, Cr, and Hg [98]. Home gardens in Kielce, Poland exhibited high concentrations of As, Cd, and Zn due to contaminated compost and chemicals, such as fertilizers and pesticides. Elevated levels of Pb and Zn were linked to the region’s natural geology and parent materials [82].

#### Best Management Practices.

Effective urban gardening in contaminated areas requires a thorough understanding of soil testing, identifying common sources of heavy metal(loid)s, and selecting appropriate crops. In urban environments, where contamination is often prevalent, gardeners should prioritize soil testing to assess risks. Once contamination is identified, several practices can reduce exposure and enhance the safety of gardening. One effective strategy is using raised beds filled with clean, imported soil. Gardeners should also focus on managing soil pH and moisture levels and keeping bare soil covered to prevent contamination [97,99,103,112]. However, raised beds should not be constructed with treated wood, as it can introduce harmful levels of As and Cu into the soil [103,111]. In fact, Clarke et al. (2015) found that 68% of soils from plots with treated wood exceeded regulatory limits for arsenic, compared to only 30% in plots without treated wood.

The placement of urban gardens is another critical factor in mitigating contamination. Traffic is a significant source of heavy metals such as Cu, Pb, and Zn in soils. Ideally, gardens should be located at least 50 meters away from street sides or shielded by physical barriers, such as dense vegetation, to reduce exposure to traffic-related pollutants [79,111].

While soil amendments can improve soil health, they can also introduce contaminants if not carefully selected. Common amendments like manure, wood ashes, and municipal compost may contain high concentrations of heavy metal(loid)s [103,112]. To mitigate contamination risks, gardeners should choose amendments that help stabilize metals. For instance, maintaining soil pH between 6.5 and 7.0 through the addition of lime can immobilize heavy metal(loid)s and reduce their bioavailability [99,111,112]. Adding organic matter, such as compost, can further decrease metal bioavailability by binding soil particles and minimizing erosion. Maintaining adequate soil moisture and covering bare soils with mulch or vegetation also reduces the risk of heavy metal exposure [97,99,112].

Proper education on contamination sources is essential, particularly in understanding which crops are more likely to accumulate heavy metals. Leafy greens and root vegetables, such as carrots, radishes, and onions, are especially prone to contamination compared to fruiting vegetables [101,112]. Regardless of the type of crop, all fruits and vegetables should be thoroughly washed before consumption to remove soil particles that may carry contaminants [100,101,111]. These proactive gardening practices can greatly reduce exposure to dangerous heavy metal(loid)s, benefiting not only urban gardeners but also nearby residents.

#### Environmental risk assessment.

Environmental indices (Table 5, Fig 6) reveal that although PERI indicates low ecological risk from heavy metals in Lafayette’s urban soils, the PLI signals notable anthropogenic contamination, with an average value of 2.14 and a maximum of 53.4. The PI results further clarify these trends. Lead and Zn pose the highest contamination risks, both exceeding the PI pollution threshold (PI > 5). There values are comparable to those reported in other contaminated urban centers, such as Brno, Czech Republic (Pb = 8.6; Zn = 10.7) [1] and Torino, Italy (Pb = 7.5; Zn = 2.9) [59]. However, Lafayette’s Pb PI remains below the extremely high levels found in Yerevan, Armenia (Pb = 22.9; Zn = 3.3) [113]. For other metals, Lafayette’s PI values (~1.5) indicate low to moderate contamination, consistent with background concentrations in urban soils of Torino, Italy (Cu = 3.3; Cr = 2; Ni = 2.8) [59] and Yerevan, Armenia (As = 1.1; Cu = 2.6; Cr = 1.7; Ni = 2) [113] The PI for Ni (0.93) falls below 1, suggesting minimal enrichment and a likely geogenic origin from regional sediments in southern Louisiana. In contrast, soils from sites between New Orleans and Baton Rouge exhibit lower contamination, with Pb PI = 1.7 and As PI < 1, indicating unpolluted conditions [114]. Moscow’s recreational soils, sampled from parks near major thermal power and waste incineration plants, also showed “unpolluted” PLI values below 1.0 for Pb, As, Cr, Zn, and Ni [115].

The highest Igeo values were calculated for Zn (6.48) and Mn (6.11), classifying these as “extremely contaminated.” Pb (2.22) and Ni (2.08) indicate “moderate to strong contamination,” while Cu (0.95) and As (−0.70) reflect minor enrichment or near-background levels. Comparative data show that Lafayette’s Zn Igeo (6.48) far exceeds values reported for urban soils in Brno, Czech Republic (Zn = 2.58) [1] and and Hangzhou, China (Zn = 0.5) [88], suggesting localized Zn accumulation, likely linked to vehicular abrasion. In cities of South India, Igeo values are generally much lower (Ni = −2.37; Zn = −1.88; Cr = −0.34; Cu = −0.07; Pb = 0.59) [116] The Igeo values for the same metals in Moscow were almost always below zero, except for Zn and As, which reached moderate levels (Igeo 1–2) only in isolated spots near industry [115]. Similarly, average Igeo values for sites between New Orleans and Baton Rouge were below zero, implying they are unpolluted with respect to Pb and As. Lafayette is classified as a low-to-medium-contamination urban environment with dominant legacy and geogenic sources rather than active pollution sources.

#### Environmental injustice: implications for public health.

The 2022 Louisiana Health Report Card documents pronounced health disparities for Black and Hispanic populations in Louisiana, especially in Lafayette Parish, where rates of diabetes, heart disease, and several cancers, including lung and colorectal, are elevated among Black adults [117]. These disparities are linked not only to socioeconomic factors but also to increased exposure to environmental pollutants such as contaminated soils and dust. Proximity to industrial zones and busy roadways further correlates with higher instances of asthma and respiratory disease, reinforcing connections between pollution and adverse health outcomes [118,119].

Although direct on soil pollution and health in Lafayette is limited, evidence from New Orleans shows that neighborhoods with predominantly Black residents, often with lower incomes, face significantly higher soil lead concentrations and related health risks compared to predominantly White neighborhoods [120]. These findings highlight a wider pattern of environmental injustice that likely extends to Lafayette, underscoring the need to investigate whether similar disparities in soil contamination contribute to local health outcomes.

Our prior mapping of legacy Pb contamination in Lafayette demonstrates higher Pb levels frequently coincide with lower-income and minority neighborhoods [29], pointing to environmental injustice in urban Louisiana. Data on other metals, notably Cd and As, further support associations with cardiovascular disease and hypertension (S3 Table) [121]. While these relationships do not prove causality in Lafayette, they reinforce the necessity for research into health effects in communities exposed to elevated soil contaminants.

Concerns about Pb exposure from older housing and hotspots, especially in areas with aging infrastructure [29], highlight the urgent need for targeted remediation and public health action. Obesity and metabolic disorders, particularly among Black and Hispanic residents, are further exacerbated by environmental stressors, amplifying the importance of addressing environmental injustice. Ultimately, focused studies that directly measure health impacts of soil pollutants, with attention to societal factors, are needed to guide effective interventions and reduce health disparities in Lafayette [29] (Heafner and Paltseva, 2024a).

## Conclusion

The observed heavy metal concentrations and land-use trends in Lafayette reflect the city’s distinctive industrial and environmental history within southern Louisiana. Although Lafayette has not undergone the intensive petrochemical or port-related pollution characteristic of Baton Rouge or New Orleans, its soils still show the imprint of urbanization, transportation corridors, and historical land use. The land-use gradient of Industrial > Roads > Residential > Parks/Gardens mirrors contamination hierarchies reported in other urban areas worldwide. Lead and zinc dominate across all land uses, with the highest levels occurring in industrial and roadside soils, likely resulting from legacy vehicle emissions, construction materials, and past industrial activities, consistent with regional patterns observed elsewhere in Louisiana.

Overall, Lafayette aligns with global urban contamination profiles but lacks the extreme concentrations typical of older industrial centers. These results characterize Lafayette as a low-to-medium-contamination urban environment, where legacy urban processes and diffuse sources, rather than ongoing industrial emissions, remain the principal contributors to soil heavy metal inputs. Despite moderate enrichment levels, overall ecological risk remains low. However, the persistence of elevated Pb and Zn in residential and recreational soils highlights the need for ongoing soil monitoring and targeted remediation, especially in areas of high public exposure. These findings underscore the need to incorporate historical land-use patterns and localized contamination sources into urban soil management and risk assessment frameworks, ensuring more accurate identification of exposure hotspots and informed mitigation strategies. Future studies should explore the direct linkages between soil contamination and public health, particularly in terms of long-term exposure to heavy metal(loid)s and their cumulative effects on vulnerable populations.

## Supporting information

S1 Table Soil heavy metal(loid) concentrations (mg/kg) (n = 1,290) in Lafayette, LA and screening levels used as references (mg/kg). (PDF)

S2 Table Soil total heavy metals (mg/kg) and soil health parameters in public and residential areas in Lafayette, LA. (PDF)

S3 Table Heavy metal(loid)s commonly found in urban soils: common sources and health concerns. (PDF)
