# Supplementary material for: Urban land use impact on soil heavy metal levels in Lafayette, Louisiana (USA)
Source: PLoS One. 2026 Mar 18;21(3):e0344559. doi: 10.1371/journal.pone.0344559 (PMC12998832; doi:10.1371/journal.pone.0344559)
Supplement: S1 Table — (PDF) [file pone.0344559.s001.pdf]

S1 Table. Soil heavy metal(loid) concentrations (mg/kg) (n=1,290) in Lafayette, LA and screening levels used as references (mg/kg).

Metal(loid)	Median	Range	LOD	USEPA	LDEQ	LFT Bkgrd	LA Bkgrd	ACA
As	8	<7-263	7	0.68	12	7	5.3	1.5-2
Cr	65	<30-3,024	30	0.3	23	41	75	100
Cu	24	<15-838	15	3,100	310	20	20	55
Mn	533	<65-20,826	65	1,800	-	284	900	950
Ni	30	<30-87	30	820	160	35	-	75
Pb	27	<8-6,877	8	200	400	14	33	13
Zn	107	<12-6,776	12	23,000	2,300	37	82	70
Cd	-	<12-20	12	7.1	3.9	-	-	0.1-0.2
Hg	-	<9	9	11	2.3	-	-	0.08

LOD	XRF limit of detection (Thermo Scientific, 2024)
USEPA	Soil screening level (Pb: updated 2024, other metal(loid)s: 2023)
LDEQ	Soil screening level (2003)
LFT Bkgrd	Lafayette geochemical background (samples collected by USDA NRCS, measured at the ULL Delta Urban Soils Lab)
LA Bkgrd	Median geochemical background for soil in a small drainage basin in Louisiana (Horowitz et al. 1991)
ACA	Average crustal abundance (Wedepohl 1995)
