# Supplementary material for: Urban land use impact on soil heavy metal levels in Lafayette, Louisiana (USA)
Source: PLoS One. 2026 Mar 18;21(3):e0344559. doi: 10.1371/journal.pone.0344559 (PMC12998832; doi:10.1371/journal.pone.0344559)
Supplement: S2 Table — (PDF) [file pone.0344559.s002.pdf]

S2 Table. Soil total heavy metals (mg/kg) and soil health parameters in public and residential areas in Lafayette, LA.

	**As**	**Cr**	**Cu**	**Mn**	**Ni**	**Pb**	**Zn**
**Busy roads (n=35)**							
Mean	12	63	35	550	35	116	190
Minimum	<7	<30	<15	251	<30	14	39
Maximum	35	87	121	935	56	527	886
CV (%)	58	22	63	34	20	112	96
**Industrial (n=43)**							
Mean	21	73	37	500	36	326	334
Minimum	<7	<30	<15	146	<30	10	32
Maximum	263	425	212	976	87	6,877	3,016
CV (%)	195	79	97	31	33	341	182
**Parks (n=288)**							
Mean	11	92	26	612	33	24	168
Minimum	<7	<30	<15	<65	<30	<8	<12
Maximum	63	3,024	577	20,826	83	203	2,416
CV (%)	73	251	135	198	21	92	175
**Residential (n=763)**							
Mean	10	64	34	564	32	105	274
Minimum	<7	<30	<15	<65	<30	<8	17
Maximum	72	253	838	1,751	68	5,659	6,776
CV (%)	60	22	135	40	16	314	213
**Gardens (n=106)**							
Mean	9	59	28	566	31	45	151
Minimum	<7	<30	<15	137	<30	<8	46
Maximum	57	241	113	1,333	47	291	797
CV (%)	67	42	46	40	10	104	70
