# Supplementary material for: Urban land use impact on soil heavy metal levels in Lafayette, Louisiana (USA)
Source: PLoS One. 2026 Mar 18;21(3):e0344559. doi: 10.1371/journal.pone.0344559 (PMC12998832; doi:10.1371/journal.pone.0344559)
Supplement: S3 Table — (PDF) [file pone.0344559.s003.pdf]

S3 Table. Heavy metal(loid)s commonly found in urban soils: common sources and health concerns.

Element		References
**Arsenic (As)**<br>Atomic no. 33<br>Density: 5.78 g/cm^3	Nonessential<br>Known carcinogen	Baladi-Mood et al. 2021; Binner et al. 2022; Bowell et al. 2014; Hu and Gao 2008; Hughes et al. 2011; Kayode et al. 2021; Shen et al. 2013; Tchounwou et al. 2012; Wang et al. 2022; WHO 2012
**Sources**		
Natural:	Volcanism, geochemical processes in groundwater	
Common mineral:	Arsenopyrite (FeAsS)	
Anthropogenic:	Mining, fossil fuel combustion, metallurgy, glass production	
Current uses:	Chemotherapy cancer treatment	
Historic uses:	Paints/pigments/dyes (1860s), human and veterinary medicine (1970s), wood preservation (2003), pesticides (1993)	
**Health concerns**	Lung, liver, prostate, skin cancer<br>*Affects virtually all organ systems	
**Cadmium (Cd)**<br>Atomic no. 48<br>Density: 8.65 g/cm^3	Nonessential<br>Known carcinogen	ATSDR 2008; Khan et al. 2016; Kubier et al. 2019; Schutte et al. 2008; Tchounwou et al. 2012; Wuana et al. 2011
**Sources**		
Natural:	Volcanism, marine sedimentary rocks, phosphates, zinc ores	
Anthropogenic:	Mining, glass production, cigarettes	
Current uses:	Rechargeable Ni-Cd batteries, critical components of airplanes and oil platforms, nuclear reactors	
Historic uses:	Paints/pigments/dyes, leather tanning, medicinal	
**Health concerns**	Lung and kidney damage/cancer<br>*Even at low levels, Cd exposure can cause bone degeneration and diseases<br>*Main exposure: for smokers: cigarette smoke; for the general population: mainly food (especially crops grown on contaminated soil).	
**Chromium (Cr)**<br>Atomic no. 24<br>Density: 7.15 g/cm^3	Nonessential<br>Known carcinogen	ATSDR 2008; Oze et al. 2007; Tchounwou et al. 2012; USEPA 1984; Xu et al. 2023; Zulfiqar et al.

**Sources**		2023
Natural:	Small amount of Cr(IV) from oxidation of Cr(III) in ultramafic or serpentine rocks	
Common mineral:	Chromite ($FeCr_2O_4$)	
Anthropogenic:	Mining, metallurgy, cement production, phosphate fertilizers, cigarettes, asbestos deterioration	
Current uses:	Alloys, paints/pigments/dyes, leather tanning, plastics	
Historic uses:	Paints/pigments/dyes, leather tanning, metallurgy, decorative chrome plating	
**Health concerns**	Lung, kidney and liver damage/cancer, asthma, skin and eye irritation	

**Copper (Cu)**<br>Atomic no. 29<br>Density: 8.96 g/cm^3	Essential<br>Toxic at high concentrations	Doebrich 2009; Oorts 2012, Panagos et al. 2018; Poggere et al. 2023; Wuana et al. 2011
**Sources**		
Natural:	Weathering of igneous and sedimentary rocks	
Common mineral:	Chalcopyrite ($CuFeS_2$)	
Anthropogenic:	Mining, metallurgy	
Current uses:	Vehicle, electrical, and construction materials	
Historic uses:	Bronze Age use of copper in jewelry, tools, coins, Cu-based fungicide (1800s)	
**Health concerns**	Anemia, liver and kidney damage, gastrointestinal issues, blood disorders	
**Mercury (Hg)**<br>Atomic no. 80<br>Density: 13.6 g/cm^3	Nonessential<br>Known carcinogen	Baladi-Mood et al. 2021; Gworek et al. 2020; Tchounwou et al. 2012; Zhao et al. 2022
**Sources**		
Natural:	Degassing of the earth's crust	
Anthropogenic:	Mining, fossil fuel combustion, metallurgy, cement production, phosphate fertilizers	
Current uses:	Electrical industry, dentistry, nuclear reactors, wood processing, pharmaceutical products	
Historic uses:	Paints/pigments/dyes, batteries, medicinal, pesticides, antifungal agent in grain storage	

**Health concerns**	Neurotoxin, eye and skin irritation, kidney damage	
**Manganese (Mn)**<br>Atomic no. 25<br>Density: 7.26 g/cm^3	Essential<br>Toxic at high concentrations	Barceloux 1999; Herndon et al. 2011; Kelly and Matos 2014; Wu et al. 2022
**Sources**		
Natural:	Ancient marine sedimentary rocks	
Common minerals:	Pyrolusite (MnO_2)<br>Rhodochrosite ($MnCO_3$)	
Anthropogenic:	Fossil fuel combustion, metallurgy<br>*Most hazardous form common near power plants	
Current uses:	Alloys, batteries, electronics, pigments/paints/dyes, Mn-based fungicide	
Historic uses:	Cave paintings, decolorizing glass, black pigment for pottery	
**Health concerns**	Severe respiratory complications, nerve damage, weak muscles, disturbs the brain’s production of serotonin and dopamine	
**Nickel (Ni)**<br>Atomic no. 28<br>Density: 8.90 g/cm^3	Essential<br>Toxic at high concentrations<br>Possible carcinogen	ATSDR 1997; Cempel and Nikel 2005; Echevarria et al. 2006; El Naggar et al. 2021; Iyaka et al. 2011
**Sources**		
Natural:	Volcanism, forest fires, vegetation, weathered parent material	
Anthropogenic:	Fossil fuel combustion, metallurgy, phosphate fertilizers, tobacco smoke	
Current uses:	Alloys, batteries, catalysts, pigments/paints/dyes, leather tanning	
Historic uses:	Coins, alloys, military (weapons, ammo, machinery)	
**Health concerns**	Respiratory and immune system problems, dermatitis, some cancers	
**Lead (Pb)**<br>Atomic no. 82<br>Density: 11.3 g/cm^3	Nonessential<br>Known carcinogen and neurotoxin	Baladi-Mood et al. 2021; Binner et al. 2022; Carr et al. 2008; Després et al. 2005; Dupont-Soares et al. 2015; Lanphear et al. 2005; Li et al.
**Sources**		

Natural:	Weathering of igneous and sedimentary rocks in hydrothermal deposits	2004; Luo et al. 2012; Mielke et al. 1998, 2023; Paltseva et al. 2022; Penteado et al. 2021; Tchounwou et al. 2012; Wang et al. 2022; Zahran et al. 2013
Common mineral:	Galena (PbS)	
Anthropogenic:	3 primary sources: the historic use of Pb-based paints, leaded gasoline, and point source emitters such as smelters and battery dumpsites	
Current uses:	Lead-acid batteries, ammunitions, metal products, devices to shield X-rays	
Historic uses:	Pb-based residential paint (U.S. ban in 1978), leaded gasoline for on-road vehicles (U.S. phase-out completed in 1996), and lead in new drinking water pipes (U.S. restrictions introduced in 1986)	
**Health concerns**	**Children**: Diminished intelligence, slowed neurological development, decreased cognitive function<br>**Adults**: Adverse effects on the blood, central nervous system, kidneys, reproductive system, and gastrointestinal diseases	

**Zinc (Zn)**<br>Atomic no. 30<br>Density: 7.14 g/cm^3	Essential<br>Toxic at high concentrations	Hussain et al. 2022; Kropschot and Doebrich 2011; Noulas et al. 2018; Wuana et al. 2011
**Sources**		
Natural:	Sedimentary and granitic rock weathering	
Common mineral:	Sphalerite (Zn, Fe)S	
Anthropogenic:	Mining, fossil fuel combustion, metallurgy, cement production	
Current uses:	Galvanized metal, brass and bronze alloy, rubber, chemicals, pigments/paint/dyes, Zn-based fertilizers and pesticides, skin ointments	
Historic uses:	Brass alloy, medicinal	
**Health concerns**	Gastrointestinal distress (vomiting, nausea, cramps, diarrhea, epigastric pain)	
